# An exploratory transmission mode of HIV/AIDS among older people based on data from multiple sources in China

**DOI:** 10.1038/s41598-022-20146-2

**Published:** 2022-09-27

**Authors:** Xiwei Sun, Caibi Feng, Liao Feng, Ticheng Xiao, Xinran Zhao, Hong Liu, Zhiqiu Wang, Chen Chen, Shoulin Zhou, Dinglun Zhou

**Affiliations:** 1 West China School of Public Health and West China Fourth Hospital, Sichuan University, Chengdu, China; 2 Luzhou Center for Disease Control and Prevention, Luzhou, China; 3 Sichuan Center for Disease Control and Prevention, Chengdu, China; 4 Cornell University College of Veterinary Medicine, Ithaca, USA

**Keywords:** Public health, Epidemiology

## Abstract

The HIV/AIDS cases and proportion in older people are continuously and rapidly increasing in China, especially in males. However, the transmission mechanism is not well understood. This study aims to explore the transmission mechanism of HIV/AIDS and potential ways to prevent or control HIV/AIDS transmission in a city in southwestern China. Data from multiple sources, including HIV/AIDS case reports in 2010–2020, a survey of HIV/AIDS cases in 2020, and sentinel surveillance data of female sex workers (FSWs) in 2016–2020 were analyzed. We explored the transmission mechanism of HIV/AIDS cases aged 50 years and older. In this city, the number of newly reported HIV/AIDS cases aged 50 years and older increased from 2010 to 2019, and decreased in 2020. The number of male and female cases aged 50 years and older both increased rapidly in 2017–2019, though the number of male cases was larger than that of female. The survey data showed that 84.7% of older male cases reported commercial sexual behavior, among whom 87.7% reported never using condom and 37.6% reported more than 10 times of commercial sexual behaviors in 1 year. In terms of price of sexual behavior, 68.3% of older male cases sought low-tier FSWs among whom the HIV/AIDS positive rate was 5.4% from the sentinel surveillance data. These results suggested HIV transmission between older men and low-tier FSWs through commercial sexual behavior. Among female cases aged 50 years and older, most reported non-marital and non-commercial heterosexual (60.5%) or regular sexual partner (31.4%) transmission, suggesting that they were infected by their spouse/regular sexual partner. Data of matched couples showed that most male cases had both marital sexual behavior and commercial sexual behavior, or acquired HIV through commercial sexual behavior, while most female cases had only marital sexual behavior. Based on these findings, we proposed a transmission mode of that local older male people and FSWs are transmitting bilaterally through commercial sexual behavior, and local older male people are spreading to local older female people through spouse or regular sexual partner route. By studying data from multiple sources, we explored the HIV transmission mode among older people. In the meantime, we found that marital status had a different impact on male and female old people in their sex behaviors.

## Introduction

The prevalence of AIDS is still a huge challenge to public health around the world^1^. There were nearly 37,700,000 people globally living with HIV, 680,000 AIDS-related deaths and 1,500,000 new HIV infections in 2020^2^. In China, national estimates indicated that the number of estimated new HIV infections was around 80,000 by the end of 2018, and the number of people living with HIV (PLHIV) reached more than one million by the end of 2020, and kept growing, causing much pressure for HIV/AIDS prevention and control^3^.

In China, the United States and many other countries, HIV/AIDS was closely related to intravenous drug use (IDU) and the behavior of sharing needles in the early epidemic stage^4–6^. However, IDU has declined over time and now represents a very small proportion of PLHIV in China. The major transmission route has shifted from IDU to sexual transmission in China, including homosexual and heterosexual transmission^7–10^. The proportion of HIV/AIDS infected due to heterosexual transmission has increased from 33.1% in 2016 to 71.1% in 2018^11^.

In recent years, both at the national level and provincial level in China, the number of newly reported cases aged 50 years and older has been increasing^9,12–16^. For example, the proportion of older people in reported HIV cases increased from less than 4% in 2000 to more than 40% in 2013 in Guangxi Province, China^17–19^.

The UNAIDS has set 95–95–95 targets to combat HIV and AIDS globally, aiming that by year 2025, 95% of PLHIV would know their HIV status, 95% of those testing HIV positive would be on anti-retroviral treatment (ART), and 95% of those on ART would achieve viral load suppression^20^. Also, UNAIDS calls for ending the AIDS epidemic as a public health threat by 2030. Due to the increasing number of newly reported HIV/AIDS older cases, it is important to understand the transmission mechanisms of HIV/AIDS among older adults, especially the causes of sexual transmission. However, there are few studies on the transmission mechanism among older adults. In this study, we used data from multiple sources, including case reports of new HIV/AIDS cases, survey data of HIV/AIDS cases focusing on sexual behaviors, and sentinel surveillance data targeting FSWs, to explore the transmission mechanism among older adults.

## Methods

This study focused on the population in a city in southwestern China, which has a permanent population of 4.3 million at the end of 2020. From 2010 to 2020, a total of 14,824 HIV/AIDS patients were registered in the city, and the HIV infection rate was 0.3%. About three quarters of those infected were between 40 and 70 years old, and the mortality rate of those infected with HIV was about 24%. We studied older people aged 50 years and older living with HIV/AIDS.

### Data sources

Data from three sources were used, including cases reports of new HIV/AIDS cases, survey data of HIV/AIDS cases focusing on sexual behavior, and sentinel surveillance data of FSWs. All of these data were collected from one city in Sichuan Province, southwestern China.

#### Case reports of new HIV/AIDS cases

Data was obtained from the official website of the Chinese Center for Disease Control and Prevention (CDC). HIV infections and AIDS patients are diagnosed by the principles of HIV/AIDS diagnostics and the Chinese national HIV/AIDS test technology regulation and criteria, respectively. All cases are recorded in a real-name registration system. To avoid duplication of reported cases, regular checks are carried out by the Chinese CDC to identify duplicates and retain a single record when duplicates are detected.

#### Survey data of HIV/AIDS cases

A survey focusing on sexual behavior was conducted in 2020 by the City CDC in a random sample of newly reported HIV/AIDS cases who reported HIV/AIDS acquisition from sexual behavior. The survey collected information on sexual behavior and condom use at an average of around 10 days after diagnosis.

#### Sentinel surveillance data on FSWs

Sentinel surveillance data were collected by the City CDC on FSWs from 2016 to 2020. The data included 2000 FSWs with around 400 FSWs from each year. FSWs were classified into three tiers according to the price of sexual behavior, including high-tier defined as more than ¥ 100 RMB, middle-tier defined as ¥ 50–100 and low-tier defined as less than ¥ 50.

Women included in the Sentinel surveillance were those who self-reported sex for money within the previous 3 months, and agreed to testing and counseling for HIV and syphilis. After providing informed consent, they were asked standardized questions about their demographics, potential HIV risk factors, cognitive status and health behaviors, as well as outreach services related to HIV.

All data were collected in accordance with relevant guidelines and regulations, and consent was obtained from all participants and their privacy was guaranteed throughout the study. This study was approved by the Ethics Committee of West China Fourth Hospital and West China School of Public Health, Sichuan University (Gwll2022062).

### Statistical analysis

Data were entered into EpiData 3.1 and exported to Excel 2019 for data cleaning. Statistical analysis was performed using SPSS 18.0 with a significant level of 0.05 at two-sided. Frequencies and percentages for categorical variables were reported.

We firstly described the characteristics and transmission chain of HIV/AIDS, then studied 66 couples of HIV/AIDS cases identified through two data sources. Based on all results, we proposed a HIV/AIDS transmission model.

## Results

### Characteristics of the newly reported HIV/AIDS cases

There were 14,824 HIV/AIDS cases reported in the city from 2010 to 2020, with more male cases than females (male to female sex ratio: 2.8:1). The majority of them were aged 50 years and older (60.1%) and were peasants (66.4%). In terms of marital status, 46.1% of them were married and 35.0% were divorced/widowed.

**Figure 1:**
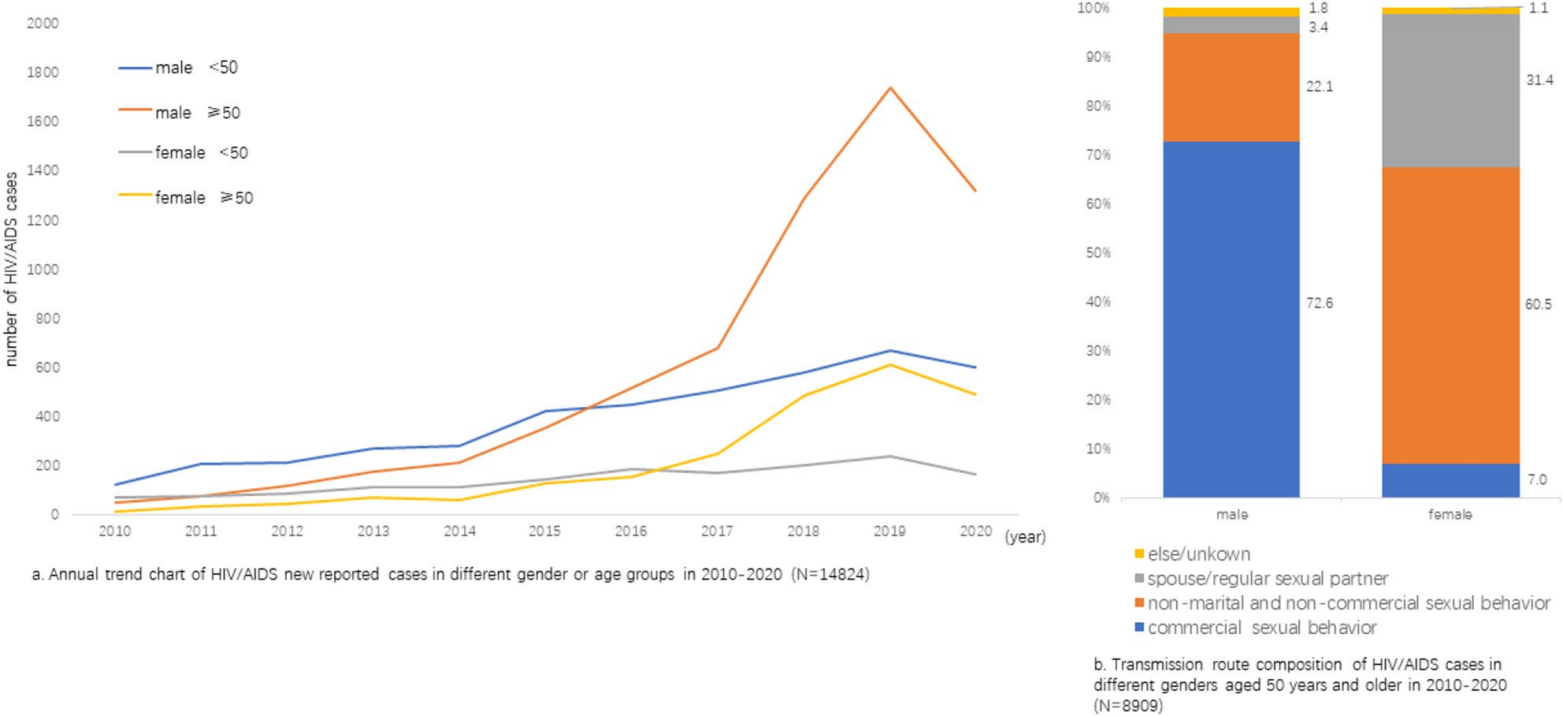
Growth trend of newly reported HIV/AIDs cases and transmission route among cases aged 50 years and older in 2010–2020.

The number of newly reported cases increased throughout the years reaching the highest in 2019 and slightly decreased in 2020 (Z = 4.0, *P* < 0.001). The growth trend of male cases aged 50 years and older appeared to be the fastest in 2017–2019 and had the largest number of cases in 2019 (Fig. 1a).

The reported routes of transmission were different between male and female (${\upchi }^{2}$ = 3403.6, *P* < 0.001). For males, the major transmission route was commercial sexual behavior (72.6%), followed by non-marital and non-commercial sexual behavior (22.1%). For females, 60.5% of cases were infected through non-marital and non-commercial sexual behavior and 31.4% were infected by spouse/regular sexual partners (Fig. 1b).

### Characteristics of male cases aged 50 years and older

There were 1383 HIV/AIDS cases surveyed in 2020. After removing 90 cases with incomplete information, 1293 were retained in analysis. There were 984 male cases and 309 female cases, and most of them were aged 50 years and older (71.2%). A majority of the cases were peasants (70.8%) married (55.6%), and having primary school education level (55.1%). Among those aged 50 years and older (n = 921), 41.8% of male cases were 60–70 years old, 54.5% of female cases were 50–60 years old, and 61.3% were married or had regular sexual partners.

**Figure 2:**
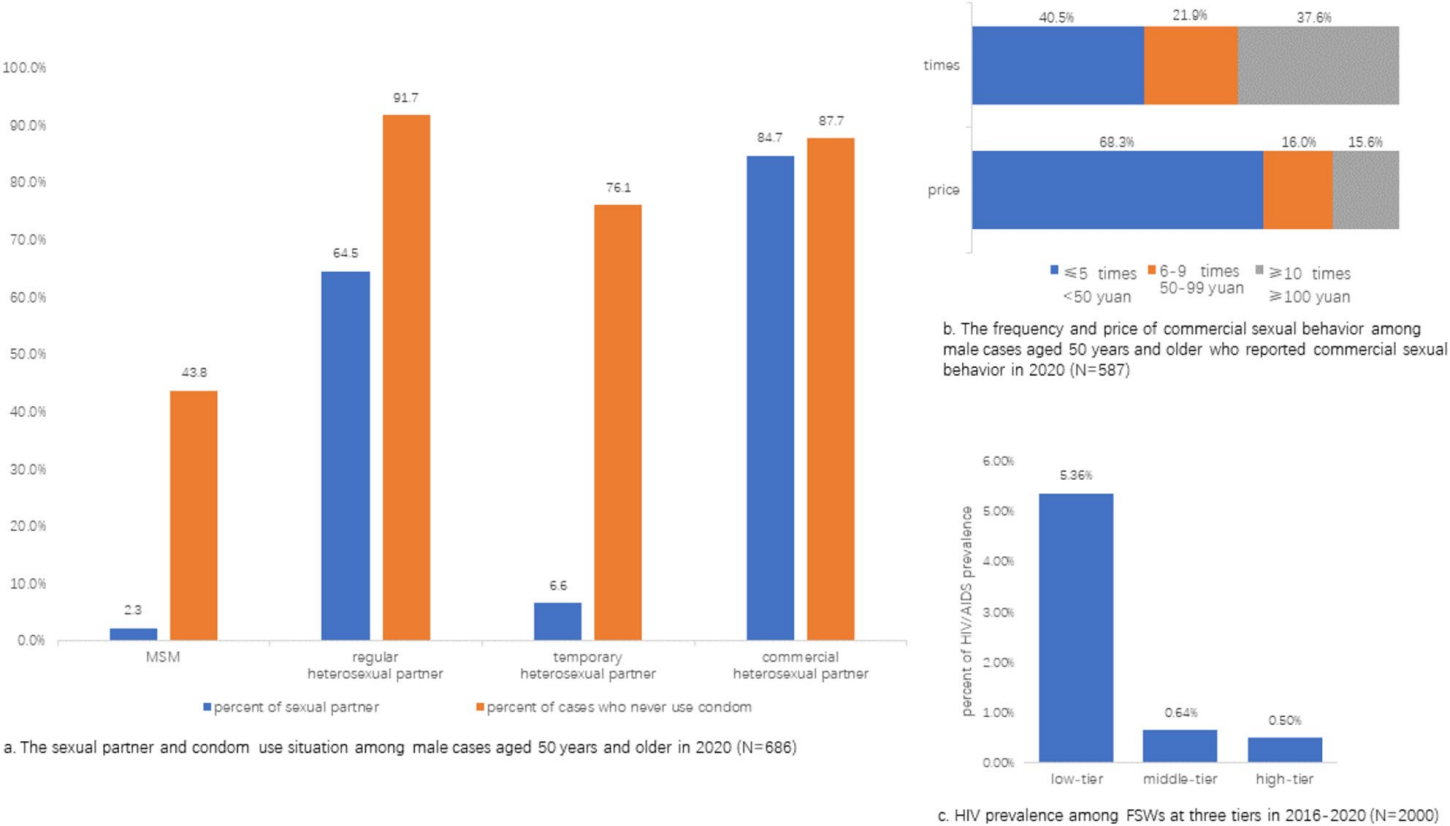
Sexual behaviors in HIV/AIDs male cases aged 50 years and older and the details of their commercial sexual behaviors.

Among 686 male cases aged 50 years and older surveyed, most of them reported commercial heterosexual partner (84.7%) or regular heterosexual partner (64.5%) (Fig. 2a). A majority of those who had regular heterosexual partner (91.7%) and those who had commercial heterosexual partner (87.7%) reported not using condom.

In the sentinel surveillance of 2,000 FSWs in 2016–2020, 821 worked in low-tier category, 780 worked in middle-tier category and 399 females worked in high-tier category. In the low-tier category, most of FSWs were 30–60 years old (88.1%), married (67.0%) or divorced/widowed (26.2%) and illiterate/having primary education (71.0%). In high-tier category, most FSWs were 30–40 years old (67.4%), married (74.7%) and having junior high school education (51.6%).

Among 587 male cases reporting commercial sexual behavior, 384 (68.3%) reported seeking low-tier FSWs and 216 (37.6%) reported commercial sexual behavior over 10 times in 1 year (Fig. 2b). Married status had no association with whether male cases had commercial sexual behavior (${\upchi }^{2}$ = 1.4, *P* > 0.1), but married males had fewer times of commercial sexual behavior than divorced/widowed or single males (${\upchi }^{2}$ = 154.1, *P* < 0.001).

Through the FSWs data, we found that the infection rate was 5.4% among low-tier FSWs, which is significantly higher than middle-tier (0.6%) and high-tier FSWs (0.5%) (Fig. 2c).

### Characteristics of female cases aged 50 years and older

**Figure 3:**
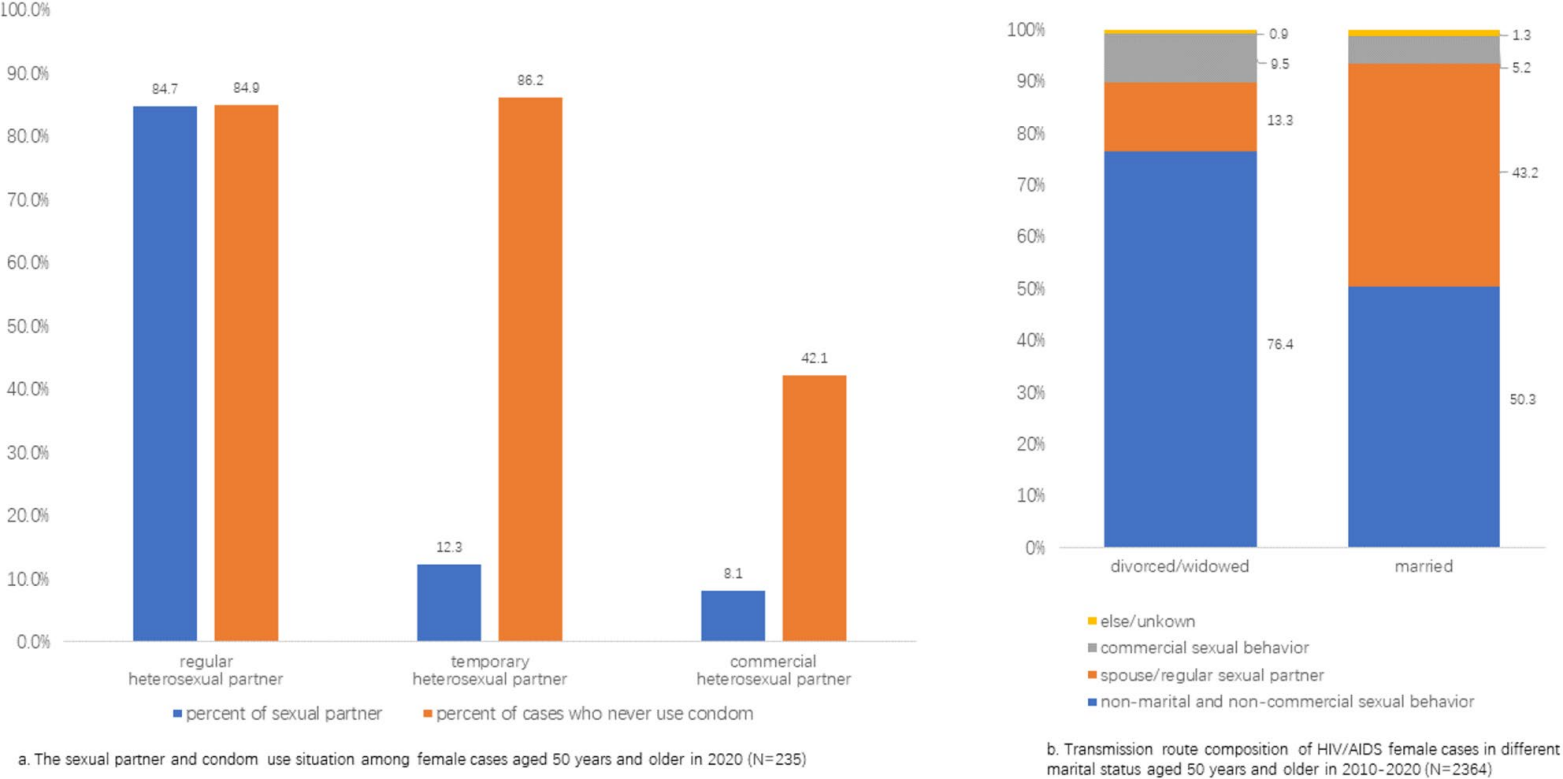
Sexual behavior of female cases aged 50 years and older and the composition of transmission routes.

For female cases aged 50 years and older that were surveyed, most of them reported only one type of sexual behavior (74.9%). There were 84.7% of female surveyed cases reporting regular sexual partners and 84.9% reporting never use condom (Fig. 3a).

A majority (76.4%) of divorced/widow women cases aged 50 years and older and 50.3% of married ones were infected through non-marital and non-commercial heterosexual behavior. The proportion of women infected through spouse or regular sexual partner route was 43.2% in married women and 13.3% in divorced/widow women (Fig. 3b).

### Additional findings of transmission chain based on linked data

There were 619 cases both of whom and their spouses were HIV/AIDS positive, including 513 cases whose spouses were also reported in the case reports in the CDC data. Among these cases, 70 couples have participated in the survey in 2020. We further identified 66 couples who reported sex behavior with their spouses recently for the analysis on transmission chain.

**Figure 4:**
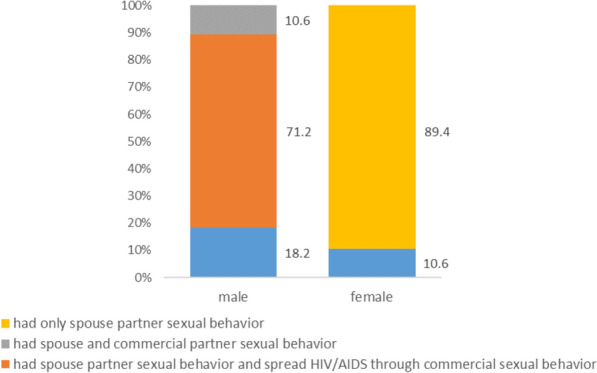
Sexual behaviors of 66 couples who had sexual behavior with spouse (N = 66).

Among the 66 married female cases, 59 (89.4%) reported only having had sex behavior with their spouses. On the other hand, among the 66 husbands, 47 (71.2%) reported commercial sexual behavior while having sex with their spouses; 7 cases (10.6%) reported no commercial sexual behavior recently but were infected through commercial sexual behavior (Fig. 4).

These results showed that while married male cases who had sexual behavior with their spouses/partners also had commercial sexual behavior frequently, married female cases had sexual behavior almost exclusively with their spouses, suggesting that married female cases were mainly transmitted by their spouses.

### A proposed mode of HIV/AIDS transmission based on data from multiple sources

**Figure 5:**
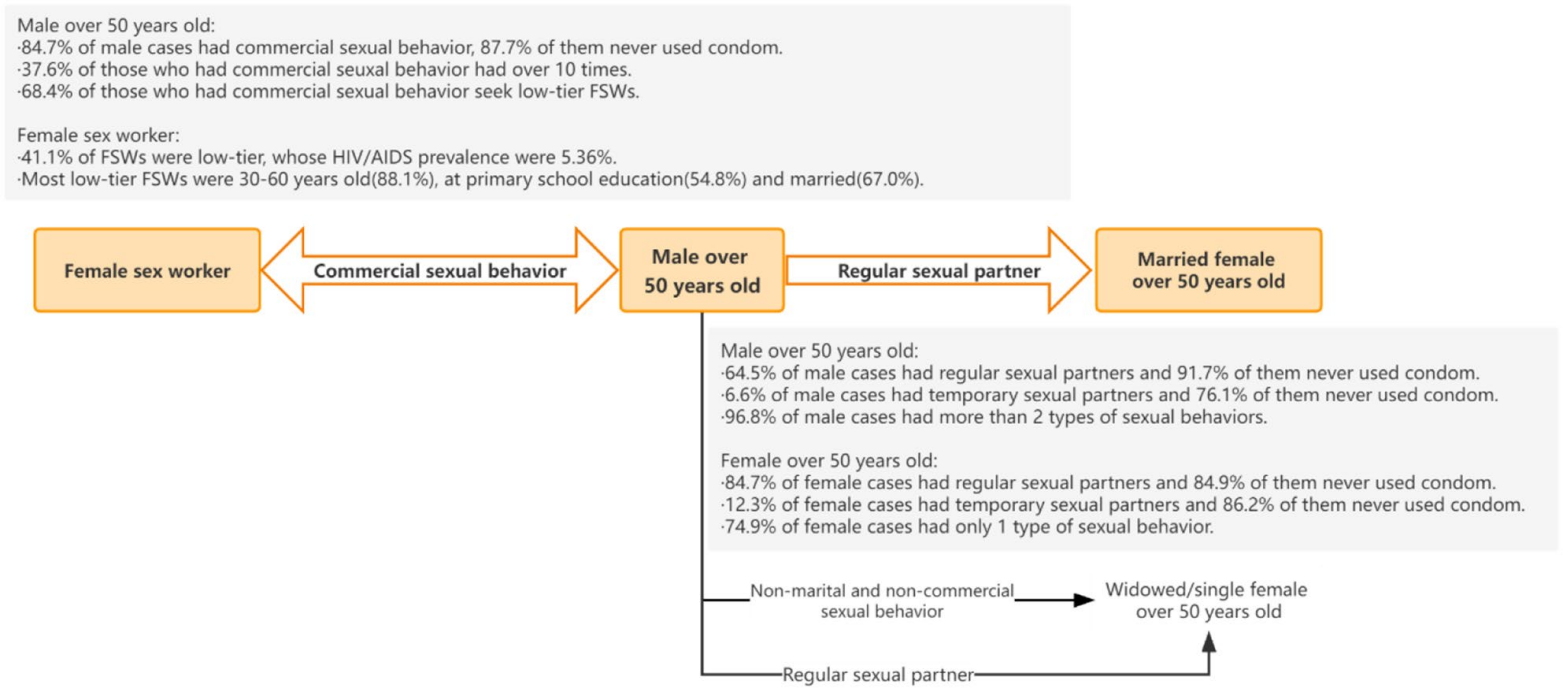
A mode of transmission based on data from multiple sources.

With the above findings, we proposed an HIV/AIDS transmission mode of local HIV/AIDS epidemic (Fig. 5). As illustrated in the mode, due to the fact that males aged 50 years and older frequently had commercial sexual behavior and they tended not to use condoms, there was a mutual transmission route between men aged 50 years and older and FSWs.

Among male cases, there was a high rate of having non-marital and non-commercial partners, and the rate of having spouse/regular sexual partner was high; marital status was not associated with the status or frequency of the incidence of commercial sexual behavior. On the other hand, the rate is also high for those who had non-marital and non-commercial heterosexual behavior and spouse or regular sexual partner among females. These findings would explain the rapid increase of female HIV/AIDS cases aged 50 years and older.

## Discussion

While there were many studies on the increase of AIDS among older people and the main transmission route of heterosexual behavior^9,21^, the reasons that male cases rapidly increased and the transmission route are not well understood, especially in male cases aged 50 years and older^22^.

In this study, we found that the proportion of HIV/AIDS among older people was increasing rapidly, and males accounted for the majority. Although the number of women was smaller, there was also an increasing trend in 2017–2019. The epidemic trend in this city seemed consistent with those found in many studies, both at national level and in regional area^19,22–24^. As we all known, sexual transmission has already become the predominant route of HIV infection in China^25^. In our study, local transmission was mainly through heterosexual sex behavior. Furthermore, we found differences in transmission routes between males and females based on data from multiple sources. Local older male people and FSWs are transmitting bilaterally through commercial sexual behavior, and local older male people are spreading to local older female people through spouse or regular sexual partner route.

Our study showed that the local prevalence of HIV/AIDS in low-tier FSWs was significantly higher than that in middle/high-tier FSWs, and also higher than those reported in other studies^26–29^. In our study, the newly reported male cases were mainly having primary school education and peasants, and the price of commercial sex was low. Most of the low-tier FSWs are middle-aged married or divorced/widowed females and at primary school education. The characteristics of the two groups indicated that the commercial partners of elderly men are mainly low-level FSWs, which is consistent with findings in other studies^9,23,30–32^. Other studies have also found that the male clients of FSWs were bridge population from FSWs to low-risk general population^32–37^. In Guangxi, although the rate of infection among FSWs decreased significantly from 2010 to 2015, the rate of infection among older clients of FSWs was still increasing^19^. In combination with the facts that many male cases reported 10 times or more of commercial sex behaviors a year and seldom use of condoms, we hypothesized that older male people and low-tier FSWs are spreading each other and driving up the local HIV epidemic synchronously.

In the meantime, we found that although female cases are fewer than male cases, the increasing trend of them was rapid as well. Marital status seemed to be playing an important role. About half of married female cases have non-marital and non-commercial sexual behavior as well as regular sexual behavior, while in divorced/widowed female cases, only about one-third of them had non-marital and non-commercial sexual behavior and about one-tenth of them had regular sexual behavior. The rate of never using a condom was very high in both regular and temporary heterosexual behaviors. At the same time, local male cases had a high rate of reporting non-marital and non-commercial partners, as well as a high rate of having spouse/regular sexual partner, which indicated that the older males also transmitted HIV to older females through regular sexual behavior and non-marital and non-commercial sexual behavior. These facts explain that the number of female older cases was fueled by a rapid increasing trend as well^26,38^.

Additional result was found that the transmission route was different between men and women. In males, while marital status did not seem to affect the incidence of commercial sexual behavior, married males had fewer times of commercial sexual behavior than divorced/widowed or single males in the recent year (Fig. 2), which is consistent with findings from another study^22^. However, marital status did not affect the source of infection in women in this study, which is different from the above study^22^. In addition, marital status affects the gender constitution of the different categories of sexual behavior. Studies show that women are more serious in intimate relationships, but men tend to seek companionship from others to solve their own loneliness^22^. In emotional relationships, women are more inclined to seek permanent partners, but may be impacted due to the other one being reluctant to use condoms; on the other hand, men tend to engage in commercial or temporary sex and use condoms irregularly. This supports the findings in this paper that women were more likely to be infected through regular sexual partner/non-marital and non-commercial sexual partner and men were more likely to be transmitted from commercial sexual partner. Other studies have shown similar results that the rate of men to women infection from men who engage in commercial sexual behavior is far higher than that from men with non-marital and non-commercial sexual behavior^22^.

The current situation of heterosexual transmission among older people is different from transmission in other groups such as MSM, which is a pattern of transmission in the general population. Therefore, it is becoming more necessary to combine the local general population with the high-risk population for analysis. In the meantime, while most of the existing studies used a single source of data, this study used data from multiple sources to explore the transmission mechanism.

This study has a few limitations. Firstly, we focused on one city in southwest China and the results may not be generalizable to other regions or countries. However, given the rapid growth of older HIV/AIDs cases both in China and world-wide, our findings have public health importance for the precise prevention and control of AIDS. Secondly, while we analyzed the three data sources together, the individual analysis of each data is not particularly detailed. Lastly, our description and comparison were mostly at group level, and there were differences in the three databases in terms of time frames, populations, and sampling modes. Therefore, it is possible the results are not homologous at individual level. We have linked social networks to the social characteristics between older male people and low-tier FSWs, and speculated that older male people and low-tier FSWs were infecting each other and driving up the epidemic in older female people at the same time. However, if evidence can be found from molecular network^39^, homology analysis or social-molecular cooperative network, it would be more beneficial to improve the evidence chain of this theory.

## Conclusion

In this study, we found that HIV/AIDS in a local city in China was increasing rapidly in older people, mainly through sexual transmission, but there are some differences between males and females.

Older male cases were mainly infected through commercial sexual behavior. The likely main reasons are that older males tended to seek low-tier FSWs, and the infection rate of low-tier FSWs was high. As males sought FSWs frequently and seldom use condoms, these two groups spreaded HIV to each other.

Older female people were mainly transmitted through regular sexual partners or temporary sexual partners. The proportion of these two types of sexual behavior was high and use of condom was irregular, which may result in the transmission from older males to females.

Based on these findings from multiple data sources, we proposed a mode of transmission, in which older males were an important bridge population spreading HIV/AIDS from FSWs to local general population. We believe that prevention and management efforts should be strengthened for men aged 50 years and older, which is crucial for preventing the spread of HIV/AIDS to the general population and controlling the AIDS epidemic.

## Data Availability

The datasets generated and/or analyzed during the current study are not publicly available due to the sensitivity of the data (we promised not to use individual patient information for analysis, but to do it at the population level), but are available from the corresponding author on reasonable request.

## References

[1] Pan SW, Li DL, Carpiano RM, Spittal PM, Ruan YH (2016). Ethnicity and HIV epidemiology research in China. Lancet.

[2] UNAIDS. *Global HIV & AIDS Statistics—Fact Sheet*. https://www.unaids.org/en/resources/fact-sheet. (2021).

[3] Lyu P, Chen FF (2019). National HIV/AIDS epidemic estimation and interpretation in China. Zhonghua Liu Xing Bing Xue Za Zhi.

[4] Aceijas C (2006). Estimates of injecting drug users at the national and local level in developing and transitional countries, and gender and age distribution. Sex. Trans. Inf..

[5] Bastos FI, Caceres C, Galvao J, Veras MA, Castilho EA (2008). AIDS in Latin America: Assessing the current status of the epidemic and the ongoing response. Int. J. Epidemiol..

[6] Wang L (2015). HIV epidemic among drug users in China: 1995–2011. Addiction.

[7] Pang XW (2021). Patterns and risk of HIV-1 transmission network among men who have sex with men in Guangxi, China. Sci. Rep..

[8] Xiao CH (2020). The changing modes of human immunodeficiency virus transmission and spatial variations among women in a minority prefecture in southwest China: An exploratory study. Medicine.

[9] Yuan FS (2021). Epidemiological and spatiotemporal analyses of HIV/AIDS prevalence among older adults in Sichuan, China between 2008 and 2019: A population-based study. Int. J. Infect. Dis..

[10] Xing JN (2014). HIV/AIDS epidemic among older adults in China during 2005–2012: Results from trend and spatial analysis. Clin. Infect. Dis..

[11] National Center for AIDS/STD Control and Prevention CC (2018). update on the AIDS/STD epidemic in China the third quarter of 2018. Chin. J. AIDS STD.

[12] O'Keefe KJ, Scheer S, Chen MJ, Hughes AJ, Pipkin S (2013). People fifty years or older now account for the majority of AIDS cases in San Francisco, California, 2010. AIDS Care.

[13] Liu ZQ (2018). Changing epidemiological patterns of HIV and AIDS in China in the post-SARS era identified by the nationwide surveillance system. BMC Infect. Dis..

[14] Negin J, Cumming RG (2010). HIV infection in older adults in sub-Saharan Africa: Extrapolating prevalence from existing data. Bull. W.H.O..

[15] Gebo KA (2009). HIV infection in older people. BMJ.

[16] Lazarus JV, Nielsen KK (2010). HIV and people over 50 years old in Europe. HIV Med..

[17] Wang LY, Qin QQ, Ge L, Ding ZW, Cui Y (2016). Characteristics of HIV infections among over 50-year-olds population in China. Zhonghua Liu Xing Bing Xue Za Zhi.

[18] Jiang J, Fan Q, Zhang J, Luo M, Qiu L (2020). A geographic hotspot and emerging transmission cluster of the HIV-1 epidemic among older adults in a rural area of eastern China. AIDS Res. Hum. Retrovir..

[19] Chen H (2019). HIV epidemiology and prevention in southwestern China: Trends from 1996–2017. Curr. HIV Res..

[20] UNAIDS. *2025 AIDS Targets: The Next Generation of Goals for the Global AIDS response*. https://www.unaids.org/en/resources/presscentre/featurestories/2021/july/20210721_2025-aids-targets (2021).

[21] Wang YJ (2020). Changing trends of HIV, syphilis, and hepatitis C among male migrant workers in Chongqing, China: Nine consecutive cross-sectional surveys, 2010–2018. Int. J. Environ. Res. Public Health.

[22] Dong ZL, Ma LY, Cai C, Gao GF, Lyu F (2021). Demographic features of identified PLWHA infected through commercial and nonmarital noncommercial heterosexual contact in China from 2015 to 2018: A retrospective cross-sectional study. BMC Infect. Dis..

[23] Wu XH (2017). HIV infections among older male clients of low-cost commercial sex venues in southern China. Int. J. Sex. Health.

[24] Wu JJ (2019). Phylogenetic analysis highlights the role of older people in the transmission of HIV-1 in Fuyang, Anhui Province, China. BMC Infect. Dis..

[25] Wu ZY (2018). Characteristics of HIV sexually transmission and challenges for controlling the epidemic in China. Zhonghua Liu Xing Bing Xue Za Zhi.

[26] Shannon K (2018). The global response and unmet actions for HIV and sex workers. Lancet.

[27] Ruan YH (2006). Syphilis among female sex workers in southwestern China: Potential for HIV transmission. Sex. Transm. Dis..

[28] Baral S (2012). Burden of HIV among female sex workers in low-income and middle-income countries: A systematic review and meta-analysis. Lancet Infect. Dis..

[29] Zhang L (2015). A systematic review and meta-analysis of the prevalence, trends, and geographical distribution of HIV among Chinese female sex workers (2000–2011): Implications for preventing sexually transmitted HIV. Int. J. Infect. Dis..

[30] Kim HY, Choe HS, Lee DS, Yoo JM, Lee SJ (2019). Sexual behavior and sexually transmitted infection in the elderly population of South Korea. Investig. Clin. Urol..

[31] Tang ZZ (2014). Aphrodisiac use associated with HIV infection in elderly male clients of low-cost commercial sex venues in Guangxi, China: A matched case-control study. PLoS ONE.

[32] Wang HB (2009). Prevalence and predictors of HIV infection among female sex workers in Kaiyuan City, Yunnan Province, China. Int. J. Infect. Dis..

[33] Huang ZJ (2011). "Bridge population": Sex workers or their clients?—STI prevalence and risk behaviors of clients of female sex workers in China. AIDS Care.

[34] Jin X (2010). HIV prevalence and risk behaviors among male clients of female sex workers in Yunnan, China. J. Acquir. Immune Defic. Syndr..

[35] Pan SM, Parish WL, Huang YY (2011). Clients of female sex workers: A population-based survey of China. J. Infect. Dis..

[36] Zhu J (2019). HIV prevalence and correlated factors among male clients of female sex workers in a border region of China. PLoS ONE.

[37] Sheng L, Cao WK (2008). HIV/AIDS epidemiology and prevention in China. Chin. Med. J..

[38] Orel NA, Spence M, Steele J (2005). Getting the message out to older adults: Effective HIV health education risk reduction publications. J. Appl. Gerontol..

[39] Dong XL, Wang JG, Hu BB, Liu XY (2019). Female sex workers in HIV/AIDS prevention: A social network analysis perspective. Physica A Stat. Mech. Appl..

